# The availability of non-instrumental information increases risky decision-making

**DOI:** 10.3758/s13423-023-02279-1

**Published:** 2023-04-10

**Authors:** Julian R. Matthews, Patrick S. Cooper, Stefan Bode, Trevor T.-J. Chong

**Affiliations:** 1 Turner Institute for Brain and Mental Health, Monash University, Clayton, Victoria 3800 Australia; 2 RIKEN Center for Brain Science, Wakō-shi, Saitama, 351-0198 Japan; 3 Melbourne School of Psychological Sciences, The University of Melbourne, Parkville, Victoria 3010 Australia; 4 Department of Neurology, Alfred Health, Melbourne, Victoria 3004 Australia; 5 Department of Clinical Neurosciences, St Vincent’s Hospital, Fitzroy, Victoria 3065 Australia

**Keywords:** Decision-making, Risk, Reward, Uncertainty, Information

## Abstract

Contemporary models of decision-making under risk focus on estimating the final value of each alternative course of action. According to such frameworks, information that has no capacity to alter a future payoff (i.e., is “non-instrumental”) should have little effect on one’s preference for risk. Importantly, however, recent work has shown that information, despite being non-instrumental, may nevertheless exert a striking influence on behavior. Here, we tested whether the opportunity to passively observe the sequence of events following a decision could modulate risky behavior, even if that information could not possibly influence the final result. Across three experiments, 71 individuals chose to accept or reject gambles on a five-window slot machine. If a gamble was accepted, each window was sequentially revealed prior to the outcome being declared. Critically, we informed participants about which windows would subsequently provide veridical information about the gamble outcome, should that gamble be accepted. Our analyses revealed three key findings. First, the opportunity to observe the consequences of one’s choice significantly increased the likelihood of gambling, despite that information being entirely non-instrumental. Second, this effect generalized across different stakes. Finally, choices were driven predominantly by the likelihood that information could result in an earlier resolution of uncertainty. These findings demonstrate the importance of anticipatory information to decision-making under risk. More broadly, we provide strong evidence for the utility of non-instrumental information, by demonstrating its capacity to modulate primary economic decisions that should be driven by more motivationally salient variables associated with risk and reward.

**Supplementary Information:**

The online version contains supplementary material available at 10.3758/s13423-023-02279-1.

A rich tradition in psychology and economics focuses on the cognitive processes involved in decision-making under risk. Traditional frameworks evaluate risk as a function of the subjective utility that individuals place on each course of action (Kahneman & Tversky, 1979; Samuelson, 1947). Importantly, in real life, the outcome of a risky decision is typically not immediately known. For example, a lottery must be played out before its outcome is realized. However, the intervening events (e.g., the drawing of numbers) should be irrelevant because, although informative, they cannot alter the outcome of the choice already made. Current frameworks of risky decision-making are primarily focused on the computations and statistical properties of outcomes. However, recent research shows that information itself is intrinsically valuable, even if it cannot alter future events (i.e., is “non-instrumental”; Goh et al., 2021; Sharot & Sunstein, 2020). An important question, therefore, is whether risky behavior can be modulated by the opportunity to receive information about the consequences of a decision, despite that information appearing after the decision is made, and therefore having no possible influence on one’s prior evaluation of risk.

Recent work has shown that individuals actively pursue information, even when it is devoid of instrumental utility. The paradigms utilized by most studies share two common features. First, participants are always required to make explicit choices that are singularly focused on the information itself. For example, a common approach is to directly ask individuals how curious they are about a particular outcome (e.g., Bromberg-Martin & Hikosaka, 2009, 2011; Charpentier et al., 2018; Jezzini et al., 2021; Kelly & Sharot, 2021; Kobayashi & Hsu, 2019; Liew et al., 2022; van Lieshout et al., 2018; Vasconcelos et al., 2015). Another approach is to ask participants how much of a particular commodity they are willing to sacrifice in return for non-instrumental information—for example, food (Embrey et al., 2020), water (Blanchard et al., 2015), time (Iigaya et al., 2016), money (Bennett et al., 2016; Brydevall et al., 2018; Cabrero et al., 2019), effort (Goh et al., 2021), or even pain (Lau et al., 2020). Such studies have clearly demonstrated the utility of non-instrumental information, but in the context of decisions that are solely about the properties of the information itself. In contrast, a much stronger challenge is to determine whether such information can indirectly influence separate decisions, particularly those that should be driven by more motivationally salient variables.

A second feature common to most paradigms is that participants must choose between an option that provides either complete and accurate knowledge about a future outcome, or no additional information at all (Bennett et al., 2016; Brydevall et al., 2018; Goh et al., 2021; Iigaya et al., 2020; Jezzini et al., 2021; Kobayashi & Hsu, 2019; Lau et al., 2020; Liew et al., 2022; van Lieshout et al., 2018). This work has led to frameworks which suggest that the utility of information arises, at least in part, through its capacity to reduce uncertainty, or definitively resolve it sooner than would otherwise be possible (Goh et al., 2021; Kreps & Porteus, 1978; van Lieshout et al., 2021). Notably, these two concepts are often conflated, despite being conceptually distinct. Regardless, these models predict that individuals should value even partial information, as long as it has the capacity to reduce or resolve any degree of uncertainty. However, this prediction remains to be directly tested, as most studies only probe the opportunity to reduce or resolve uncertainty in an all-or-nothing manner.

The goal of this study was to provide a strong test of the utility of non-instrumental information, by examining its capacity to modulate a primary decision that is known to be robustly governed by other motivationally salient decision variables. Decision-making under risk provides an ideal framework for this purpose. Contemporary models of economic decision-making typically estimate risk preference as a function of the statistical properties of the different alternatives—such as the distribution of possible outcomes, and the variance in their payoffs (e.g., Kahneman & Tversky, 1979; Tversky & Kahneman, 1992; Weber et al., 2004). Importantly, variables that do not directly impact on these statistical properties, or on individuals’ attitudes towards risk, are traditionally regarded as less critical. By these accounts, decision-making under risk should be relatively robust to secondary factors, such as the availability of non-instrumental information.

Here, we asked whether the opportunity to immediately observe the consequences of a risky choice affects decision-making under risk, even if this information cannot alter the outcome. Across three experiments, participants chose whether to accept or reject gambles on a five-window slot machine, which had a 50% chance of winning or losing on every trial (Fig. 1). Critically, we informed participants at the beginning of each trial which windows would subsequently provide veridical information about the outcome, should that gamble be accepted. We emphasized that this information had no bearing on the outcome of the gamble itself. By systematically manipulating the number of windows which provided veridical information about the lottery outcome, we could determine how the partial availability of non-instrumental information modulated risk-based decisions.

**Fig. 1:**
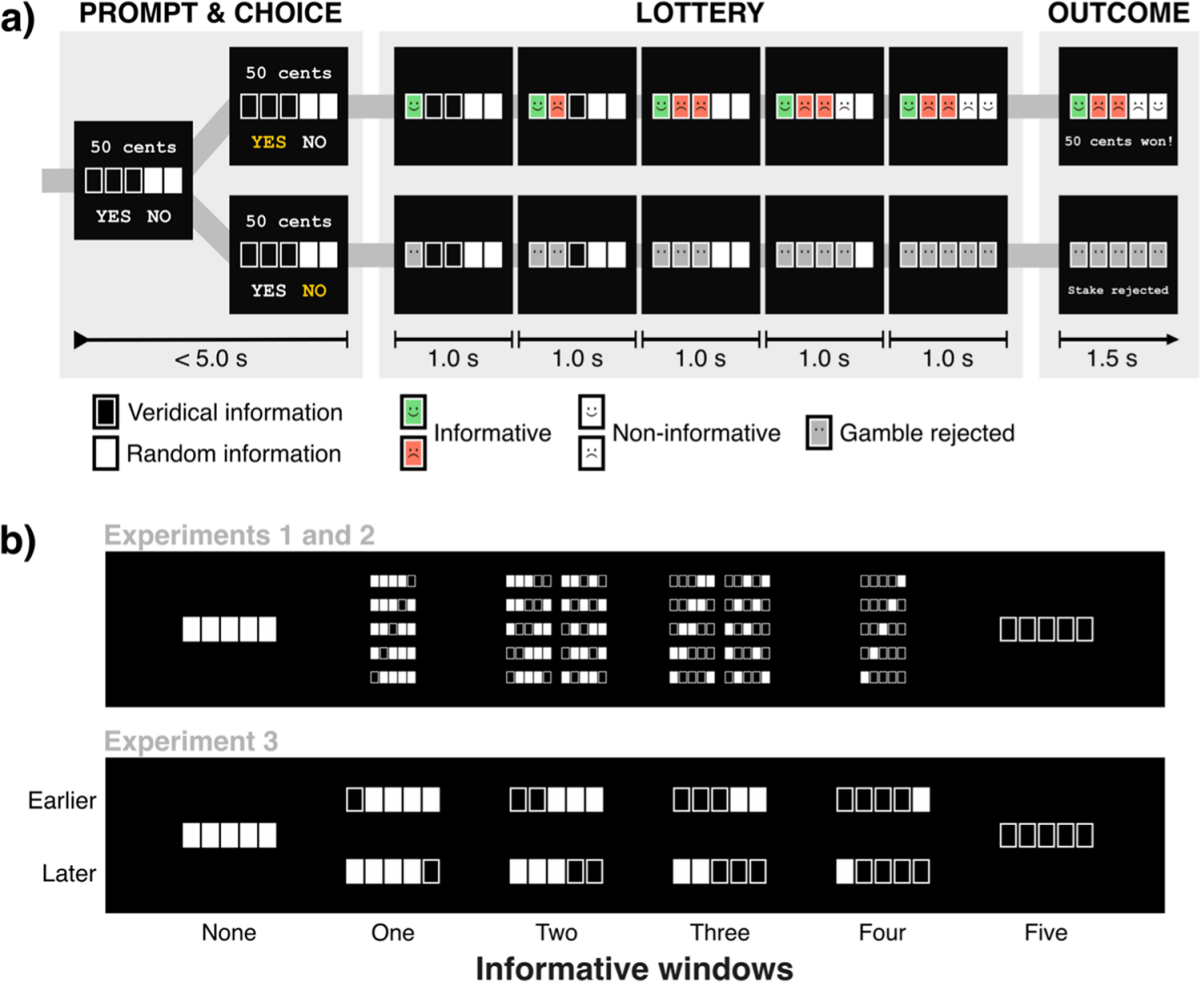
General method for Experiments 1 to 3. **a**) On each trial, participants chose whether to play a five-window slot machine, which provided an equiprobable chance of winning or losing a given stake (50¢ in Experiment 1; 10–50¢ in Experiments 2 and 3). If the lottery sequence comprised a majority of green smiley faces, participants won the amount at stake. If it comprised a majority of red sad faces, they lost that amount. The number of windows that provided veridical versus random information about the true distribution of faces ranged from 0 to 5. Regardless of the number of informative windows, the probability of winning was always 50%. Participants chose whether to accept or reject the gamble while being shown the stake and the number of informative windows. If the gamble was accepted (top row), informative windows revealed either a green smiley or red sad face. Non-informative windows revealed a random smiley or sad face. The final lottery outcome was delivered one second after the final window was revealed. If the lottery was rejected (bottom row), neutral windows (grey) with no information were displayed. Timing was identical across all trials, regardless of choice. **b**) Prompts at the beginning of each trial denoting the informative windows to be presented in the lottery. White windows depicted those that would provide random information about the lottery outcome. Black windows indicated those that would provide accurate, but non-instrumental, information about that outcome (i.e., green or red faces). In Experiments 1 and 2, arrangements were randomly selected from the options in the top panel. In Experiment 3, information appeared at either the earliest or latest possible windows in the partially informative conditions (1 to 4 informative windows). (Color figure online)

## Experiment 1

First, we established whether the opportunity to view predictive, but entirely non-instrumental, information about a lottery outcome affected the choice to gamble. Although current frameworks of risky decision-making are primarily focused on the computations and statistical properties of outcomes, recent data emphasize the utility of non-instrumental information in reducing or resolving uncertainty. We therefore predicted that the availability of such information should increase the preference to gamble.

### Method

#### Participants

In Experiment 1, we tested 22 healthy adults (12 self-identified males, 10 self-identified females, age range: 21–32 years, *M* = 23.2, *SD* = 3.2). The sample size was based on effect sizes estimated from our previous work on non-instrumental information-seeking (Brydevall et al., 2018). Note that Experiments 1 to 3 involved three separate groups of participants, and no participant engaged in any other task during their testing session. We provided participants with an endowment of $15 and informed them that their decisions on each trial would impact their final payoff. Participants were reassured that they would not leave the experiment owing the experimenter any of their own money. All participants provided written informed consent in accordance with the Declaration of Helsinki. All study protocols were approved by the Monash University Human Research Ethics Committee.

#### Apparatus

Stimuli were displayed on a CRT monitor (1,024 × 768 screen resolution; refresh rate 60 Hz). Responses were registered with the left or right arrow keys on a standard computer keyboard. The left-right response mapping was fixed for each participant but counterbalanced across participants. The experiment was presented with Psychtoolbox implemented in MATLAB (Brainard, 1997).

#### Stimuli and procedure

Participants had to decide on each trial whether to accept or reject a gamble presented on a five-window slot machine (Fig. 1a). The probability of winning on each trial was 50%. At the beginning of each trial, a prompt indicated those windows that would subsequently convey predictive information about the lottery outcome (indicated in black), and those that would not (occluded by white), should the participant accept the gamble.

If participants accepted a gamble, each window was sequentially revealed at a rate of one per second. This sequential presentation of information allowed us to evenly space the piecemeal delivery of information—an approach that has been effective in eliciting an effect of non-instrumental information (e.g., Bennett et al., 2016; Brydevall et al., 2018). Informative windows revealed veridical information about the final lottery sequence (i.e., a green smiley or a red sad face that was part of the final distribution). Non-informative windows revealed a random stimulus that did not contribute to the final sequence (i.e., a smiley or sad face, which had an equiprobable chance of being shown). One second after the final window was revealed, the true outcome of the trial was displayed for 1.5 seconds, with a correspondingly valenced auditory tone. The total delay of five seconds between choice and outcome is comparable to other recent studies on non-instrumental information (Charpentier et al., 2018; Goh et al., 2021; Iigaya et al., 2020; van Lieshout et al., 2018).

If participants chose to reject the gamble, a series of neutral faces were sequentially revealed in each window, followed by a neutral auditory tone and message (“stake rejected on this trial”). Each trial was identical in duration regardless of participants’ choices. Participants had 5 seconds to register their response, which previous studies on reward-based decision-making have demonstrated to be ample for participants to compare the value of each lottery, and indicate their preferred response (Bennett et al., 2021; Charpentier et al., 2018; Chong et al., 2017; Goh et al., 2021; Iigaya et al., 2020; Jarvis et al., 2022; Jurgelis et al., 2021). Failure to respond (a “miss”) resulted in a two second timeout, and a loss of 50¢. Misses were very rare (0.6% in Experiment 1, 0.3% in Experiment 2, 0.3% in Experiment 3; 50 misses from 12,780 trials across all experiments).

Information Availability was manipulated across six conditions from no information (zero informative windows) to full information (five informative windows). Information conditions were randomly interleaved, and participants completed 30 trials from each, for a total of 180 experimental trials. We randomly sampled across all permutations of informative and non-informative windows in the cases where there were zero, one, four and five informative windows (Fig. 1b). In the case of two and three informative windows, we randomly sampled from 10 preselected permutations. The stake for every trial was set at 50¢. Lotteries that comprised a majority of green smiley faces resulted in a win of 50¢, and those that comprised a majority of red sad faces resulted in a loss of that amount.

We emphasized that the amount of non-instrumental information did not affect the outcome of the lottery, and that the probability of winning or losing was fixed and independent on every trial (like a fair coin toss). To demonstrate that the probability of winning and losing was fixed, participants completed 15 practice trials before commencing the main experiment (information levels randomly selected). At the conclusion of each experiment, participants were debriefed to determine how they were making their decisions. Any participant who indicated that they held erroneous beliefs about the gamble probabilities was excluded (e.g., believing that the odds of winning varied from trial-to-trial). No such participants were excluded in Experiment 1. The total testing duration was approximately 60 minutes including two breaks.

#### Statistical analyses

All analyses were performed in R (R Core Team, 2022) using the lme4 and emmeans packages (Bates et al., 2015; Lenth, 2020). We used a logistic mixed model to determine the effect of Information Availability (zero to five informative windows) on Choices to accept or reject each gamble. Information Availability was the only fixed factor in Experiment 1, and was modelled as an ordinal variable to account for its natural ordering, without assuming that its effect on gambling scales linearly (although we note the pattern of results was preserved even when Information Availability was modelled as a continuous variable). Statistics were computed by conducting a Type II Wald chi-squared test on fixed factors in each model. Subject-specific intercepts were included as random effects in all models to account for individual differences in the propensity to gamble. We performed post hoc comparisons by using full models to compute the marginal mean acceptance rates for each fixed factor, and conducting Holm-adjusted pairwise contrasts.

### Results and discussion

**Fig. 2:**
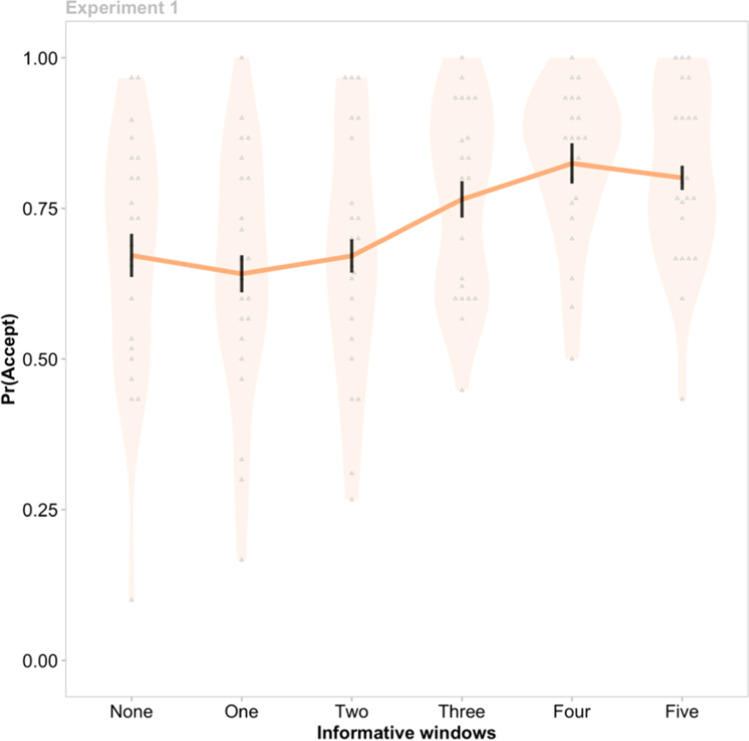
The effect of non-instrumental information on gamble acceptance rates in Experiment 1. The probability of gambling, Pr(Accept), increased as Information Availability increased between two to four windows. Black vertical lines indicate group means ±1 *SEM*. Grey points are individual participant means

This analysis revealed a significant effect of Information Availability on Choice, χ^2^(5) = 105.65, *p* < .001. Acceptance rates were similar for the conditions in which zero, one, or two informative windows were available (zero informative windows, arithmetic mean, *M* = 0.67, *SEM* = 0.04; one window, *M* = 0.64, *SEM* = 0.03; two windows, *M* = 0.67, *SEM* = 0.03; all *z*-values ≤ −1.22, p_holm_-values ≥ .89; Fig. 2). However, the willingness to gamble increased as the number of informative windows progressed from two to three windows (*M* = 0.77, *SEM* = 0.03; *z* = 3.93, p_holm_ < .001); and from three to four (*M* = 0.83, *SEM* = 0.03; *z* = 2.77, p_holm_=.03); but not from four to five (*M* = 0.80, *SEM* = 0.02; *z* = −1.13, p_holm_=.89). This result confirms that information modulates risk-based decisions, even if it has no capacity to alter future outcomes. Next, we challenged the strength of this effect by manipulating the motivational salience of the risk involved in each decision.

## Experiment 2

Several theories of decision-making under risk emphasize the importance of outcome variance in guiding choice (e.g., d’Acremont & Bossaerts, 2008; Kahneman & Tversky, 1979; Markowitz, 1952; von Neumann & Morgenstern, 1947), even when the expected values of the outcomes are held constant (Sharpe, 1964). To further challenge the utility of non-instrumental information, we manipulated risk salience by varying the amount at stake on each trial. Recent studies on non-instrumental information provide differing predictions on the effect of reward magnitude or outcome variance, with some indicating that it plays a significant role in information preference (e.g., Bennett et al., 2021), and others suggesting a less prominent role (e.g., Kobayashi & Hsu, 2019; Liew & Newell, 2021).

### Method

#### Participants

The effect of Information Availability from the logistic model in Experiment 1 (*w* = 0.78) indicated a sample size of 28 was sufficient to achieve α = .05 at power = 0.9. We tested an entirely new group of 33 healthy adults. Post-experiment debriefing revealed four participants who did not believe that the odds of winning were fixed. These participants were excluded from our analyses, leaving a final cohort of 29 (nine self-identified males, 20 self-identified females, age range: 18*-*25 years, *M* = 21.4, *SD* = 1.8). We note that incorporating the four excluded participants in the analyses did not alter the pattern of our results (see Supplementary Material).

#### Procedure

The procedure was similar to Experiment 1, except that we varied stake across five levels (10 to 50¢, in increments of 10). Importantly, this had the effect of increasing outcome variance, while holding the expected value of each gamble constant. The stake on each trial was indicated at the top of the display. Each participant completed 180 trials over the six levels of Information Availability and five levels of Stake. All other aspects of the procedure were identical to Experiment 1.

#### Statistical analyses

We implemented a logistic mixed model analysis on Choice (accept or reject), with Information Availability (zero to five informative windows), Stake (10 to 50¢), and their two-way interaction as explanatory variables. Pairwise contrasts were conducted between each condition using the same method as Experiment 1.

### Results and discussion

**Fig. 3:**
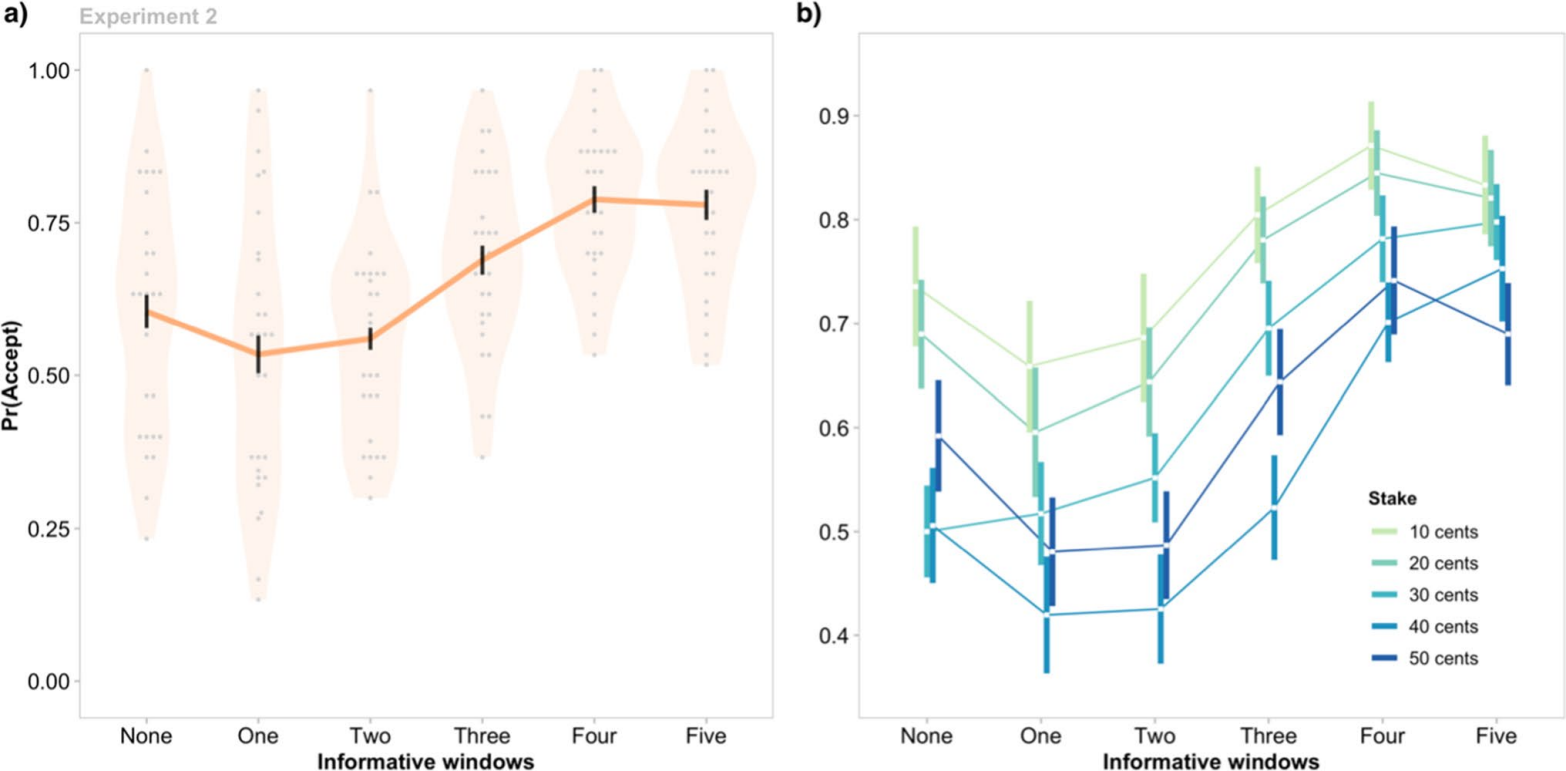
The effect of non-instrumental information and stake on gamble acceptance rates in Experiment 2. Vertical lines indicate group means ±1 *SEM*. **a)** Interestingly, the probability of gambling, Pr(Accept), was lower when one vs no informative windows were available. The increase in Pr(Accept) was greatest as Information Availability increased from two to four windows. Grey points indicate individual participant means. **b)** The effect of non-instrumental information on choice did not vary with Stake (i.e., the Information Availability × Stake interaction was not significant). (Color figure online)

The results replicated the effect of Information Availability in Experiment 1, and demonstrated that this effect was consistent across multiple Stakes (Fig. 3). Interestingly, decomposing the main effect of Information Availability, χ^2^(5) = 243.07, *p* < .001, revealed that participants accepted significantly more gambles when zero (*M* = 0.61, *SEM* = 0.03) versus one (*M* = 0.53, *SEM* = 0*.*03) informative window was available (*z* = −3.13, p_holm_ = .01). As in Experiment 1, acceptance rates were no different between one and two (*M* = 0.56, *SEM =* 0.02) informative windows (z = 1.11, p_holm_ = .526), but progressively increased as the amount of available information increased from two to three windows (*M* = 0.69, *SEM =* 0.02; *z* = 5.86, p_holm_ < .001); and three to four (*M =* 0.79, *SEM* = 0.02; *z* = 4.68, p_holm_ < .001); but not four to five (*M* = 0.78, *SEM* = 0.03; z = −0.61, p_holm_ = .55).

The main effect of Stake was significant, in a pattern that reflected risk averse behavior, χ^2^(4) = 153.16, *p* < .001. Gambles associated with lower stakes (e.g., 10¢, *M* = 0.77, *SEM =* 0.05; 20¢, *M =* 0.73, *SEM =* 0.04) were accepted more often than those with higher stakes (30¢, *M* = 0.64, *SEM =* 0.03; 40¢, *M =* 0.56, *SEM =* 0.04; 50¢, *M* = 0.61, *SEM =* 0.04; all p_holm_ < .001). Importantly, the interaction between Information Availability and Stake was not significant, χ^2^(20) = 19.87, *p* = .47 (Fig. 3b). Together, these results provide further confirmation of the effect of non-instrumental information on risky decisions, and indicate that this effect generalized across different levels of risk. Next, we consider whether the effects of non-instrumental information seen in Experiments 1 and 2 are driven by the relative timing of when that information is delivered.

## Experiment 3

In Experiments 1 and 2, the precise windows at which veridical information was to be revealed were randomly determined. However, an extensive literature on temporal discounting indicates that individuals have a strong preference for outcomes that are delivered sooner rather than later (e.g., Frederick et al., 2002; Green & Myerson, 2004). Given recent work showing that information and reward value share a common neural code (Bromberg-Martin & Hikosaka, 2011; Brydevall et al., 2018; Kobayashi & Hsu, 2019), we tested the prediction that the preference to gamble should be greater when information is delivered earlier vs later in the lottery sequence.

### Method

#### Participants

Based on the effect size of Information Availability from the logistic models in Experiments 1 (*w* = 0.78) and 2 (*w* = 1.16), a sample size of 19 was sufficient for α = .05 and power = 0.9. We therefore tested a new group of 22 healthy adults. Two participants were excluded after they indicated in post-experiment debriefing that they did not believe that the odds of winning and losing were equiprobable. The final cohort comprised a sample of 20 participants (three self-identified males, 17 self-identified females, age range: 18–32 years, *M* = 22.4, *SD* = 3.8). As in Experiment 2, incorporating the excluded participants did not alter the pattern of our results (see Supplementary Material).

#### Procedure

The procedure was similar to Experiment 2. However, for each of the partially informative conditions (one to four informative windows), we compared two possible sequences—one which provided all informative windows at the earliest possible opportunity, and another at the latest opportunity (Fig. 1b). Note that this manipulation could not be applied to conditions in which information was either complete (five informative windows) or entirely absent (zero informative windows). However, these two conditions were nevertheless included in the design to ensure that participants were making their choices within an identical reference frame to Experiments 1 and 2. All other aspects of the experiment were identical to Experiment 2.

## Results and discussion

**Fig. 4:**
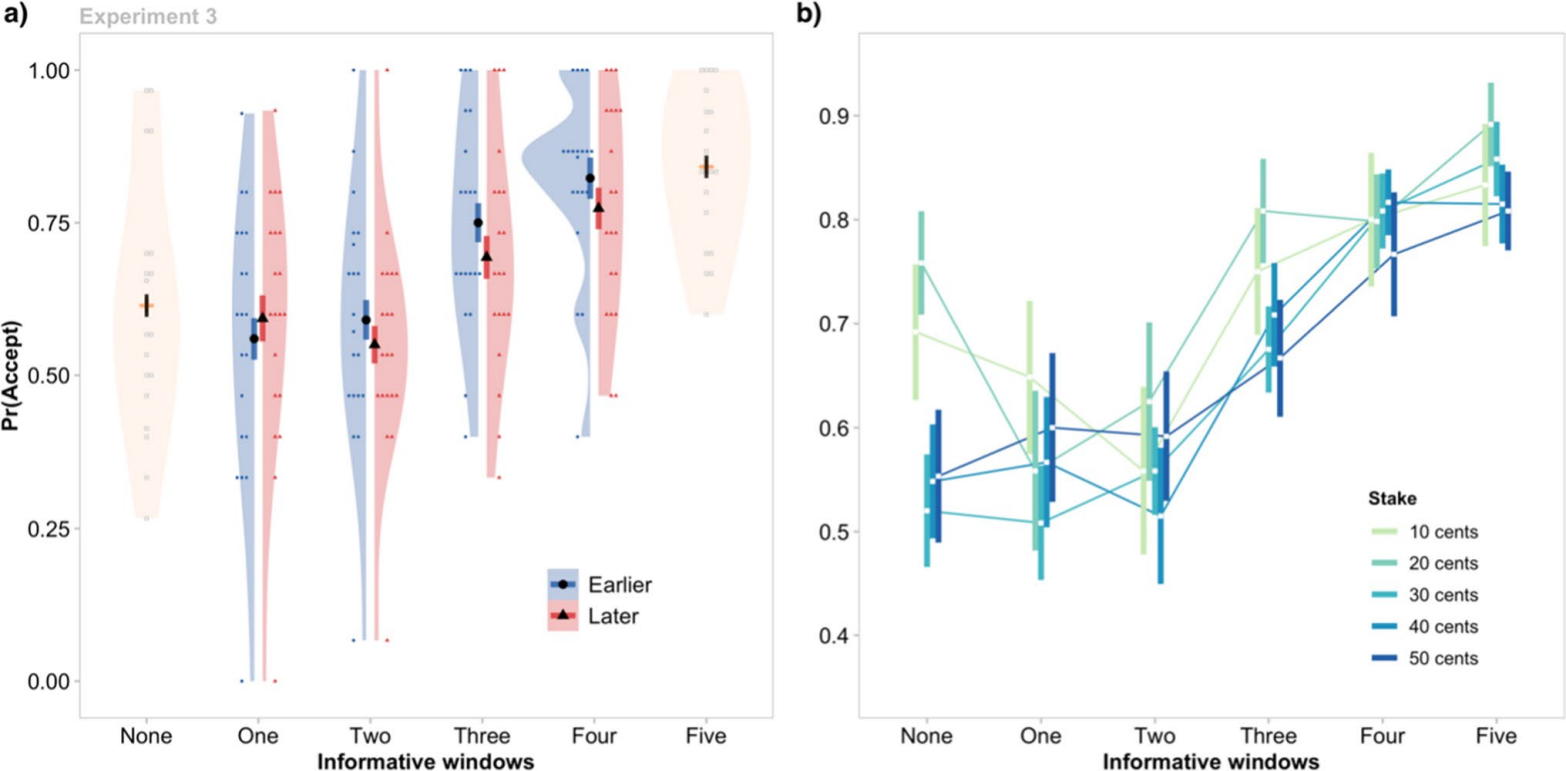
The effect of non-instrumental information, relative timing, and stake on gamble acceptance rates in Experiment 3. Vertical lines indicate group means ±1 standard error of the mean. **a)** As in Experiments 1 and 2, the greatest increase in the probability of gambling, Pr(Accept), occurred as Information Availability increased from two to four windows. When partial information was available (one to four informative windows), there was no difference in Pr(Accept) as a function of whether all information was presented at the earliest (blue) or latest (red) possible opportunities. Individual data points denote individual participant means. **b)** As in Experiment 2, the effect of non-instrumental information did not vary as a function of Stake (i.e., the Information Availability × Stake interaction was not significant). (Color figure online)

The overall findings were very similar to Experiment 2 (Fig. 4). The identical logistic mixed model revealed significant main effects of Information Availability, χ^2^(5) = 190.73, *p* < .001, and Stake, χ^2^(4) = 20.25, *p* < .001, with a non-significant interaction, χ^2^(20) = 26.12, *p* = .16. Although acceptance rates decreased between the zero-information condition (*M* = 0.61, *SEM* = 0.03), and the one (*M* = 0.58, *SEM* = 0.04) and two (*M* = 0.57, *SEM* = 0.03) window conditions, these differences were not significant (vs. one window: *z* = −1.53, p_holm_ = .252; vs. two windows: *z* = −1.77, p_holm_ = .231). The pattern of the remaining results was similar to Experiment 2. The likelihood of gambling increased at higher levels of Information Availability (three windows: *M* = 0.72_,_ *SEM* = 0.02; four windows: *M* = 0.80, *SEM* = 0.03; five windows: *M* = 0.84, *SEM* = 0.02) relative to the zero-information condition (each p_holm_ < .001). In addition, the likelihood of gambling decreased with increasing Stake (with the most pronounced differences between 20¢ and higher stakes; all *p* values < .01).

Next, we considered whether risky decisions were sensitive to the relative timing in which information was delivered, but found little evidence to support this (Fig. 4a). We examined the subset of windows in which a direct comparison of early vs late information delivery was possible (i.e., one to four windows), and performed a logistic mixed model analysis with factors of: (1) Information Availability (1–4 windows); (2) Stake (10–50¢); and (3) Relative Timing of information delivery (early vs. late); together with their full set of interactions. Most importantly, all effects involving Relative Timing were not significant (all *p* values > .113), indicating that the opportunity to receive information earlier vs later did not modulate risk-based decisions.

Although the interactions involving Relative Timing were not statistically significant, there is a visual suggestion in Fig. 4a that the acceptance rates may be higher following the early versus late delivery of information at higher levels of Information Availability. This raises the possibility that individuals may indeed prefer information when it in principle has the potential for earlier resolution of uncertainty, but that the size of any such effects may have been relatively small. To formally address this question with a more sensitive and well-powered analysis, we pooled data across all three experiments, and formally modelled participants’ choices to determine the properties of information that drove risky behavior.

### Pooled analyses

**Fig. 5:**
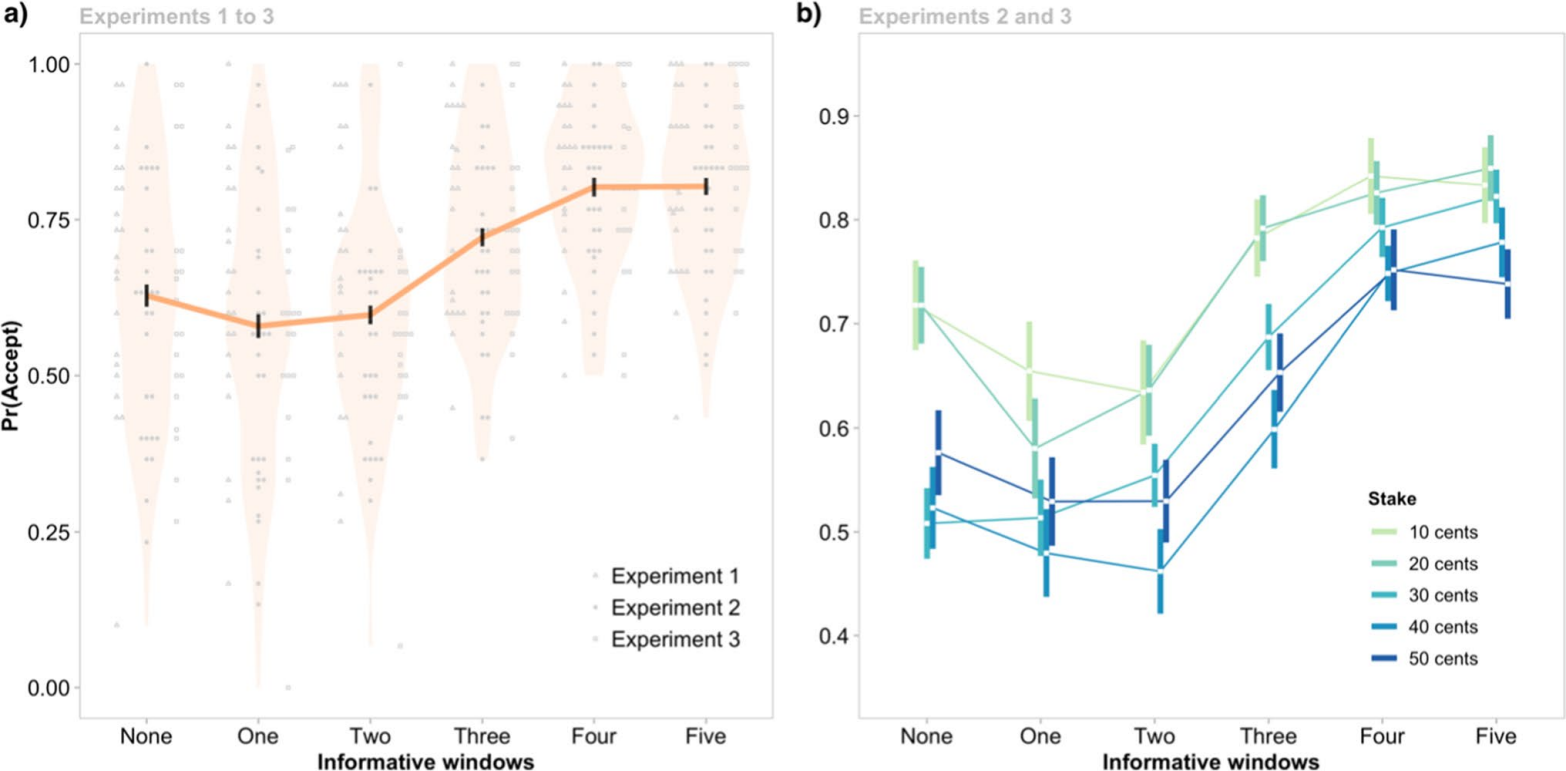
Pooled data showing the overall effect of Information Availability on the probability of gambling, Pr(Accept). Vertical lines indicate means ±1 standard error of the mean. **a)** Across Experiments 1–3, fewer gambles were accepted when there was one vs zero informative windows available. The greatest increases in Pr(Accept) occurred as the number of informative windows increased from two to four. Grey points are individual participant means. **b)** Across Experiments 2–3, the effect of Information availability on Pr(Accept) was consistent across different Stakes. (Color figure online)

First, we note that the pooled data reiterated the main effect of Information Availability (across Experiments 1–3; Fig. 5a) and Stake, with a non-significant Information Availability × Stake interaction (across Experiments 2–3; Fig. 5b). One notable feature across Experiments 1–3 was that participants accepted gambles significantly more often in the zero-information condition (*M* = 0.63, *SEM* = 0.02) than when one informative window was available (*M* = 0.58, *SEM =* 0.02; *z* = −3.39, p_holm_ = .003)—an effect that was previously only significant in Experiment 2 (Supplementary Material). For each experiment, we also performed additional control analyses to confirm that participants remained engaged throughout the task (Supplementary Material).

### Method

#### Statistical analyses

We compared four models to determine the properties of information that modulated the preference to gamble. Based on recent data, we predicted that the effect of information on risky choice should be driven primarily by either its capacity to incrementally reduce uncertainty, or definitively resolve uncertainty earlier than would otherwise occur (Bennett et al., 2016; Brydevall et al., 2018; Goh et al., 2021; Kobayashi & Hsu, 2019; van Lieshout et al., 2021). We entered pooled data for every trial for every participant across all three experiments into a logistic mixed model on Choice, with a fixed factor of “Information Value,” and subject-specific intercepts as random effects. Importantly, each model varied according to how it defined “Information Value,” as outlined below (Table 1).

**Table 1:** Information Value in four models of risky choice

Model	Number of informative windows						BIC
	Zero	One	Two	Three	Four	Five	
1. Baseline	0	0	0	0	0	0	15,120
2. Linear	0	0.2	0.4	0.6	0.8	1.0	14,703
3. Entropy reduction	0	0.104	0.228	0.392	0.625	1.0	14,683
4. Early resolution of uncertainty	0	0	0	0.25	0.625	1.0	14,643

We compared four different generalized mixed models to test how the availability of information influenced risky choice. Tabulated are the “Information Values” for every level of Information Availability, as assumed by each of the four models, and model fit results as Bayesian information criterions (BIC)

#### Model 1—Information has no effect on behavior (control model)

Model 1 was a control model which assumed that information had no effect on choice behavior. This model therefore set the Information Value of every trial to zero, and predicted choice behavior based on the subject-specific intercept alone.

#### Model 2—Information has a linear value

Model 2 postulated that the amount of information had a linear effect on choice, such that each informative window carried the same positive value. Thus, in this model, the Information Value of each trial was linearly proportional to the number of informative windows, increasing from 0 to 1.0 in equal increments of 0.2. This model differed from those in the primary analyses only in treating information as a continuous, rather than ordinal, variable.

#### Model 3—Information reduces entropy

A third model defined Information Value as the degree to which the number of informative windows could incrementally reduce uncertainty about the outcome (as opposed to the capacity of that information to resolve uncertainty definitively and unambiguously—Model 4). In information theory, uncertainty is typically quantified in terms of the Shannon entropy of beliefs (Shannon, 1948), and the information content, *I*, of a stimulus is directly related to its capacity to minimize entropy:

$${\displaystyle \begin{array}{c}I=H\left(\textrm{P}{(win)}_{prior}\right)-H\left(\textrm{P}{(win)}_{post}\right)\\ {}H=-\left\{\ \left[P(win)\bullet {\log}_2P(win)\right]+\left[\left(1-P(win)\right)\bullet {\log}_2\left(1-P(win)\right)\right]\ \right\},\end{array}}$$

where *H* is the entropy associated with the probability of winning (*P*(*win*)), and *H* = 0 when *P*(*win*)= 0 or 1.

In our study, the prior probability of winning, P(*win*)_*prior*_, was always 0.5. Thus:

$$H\left(\textrm{P}{(win)}_{prior}\right)=1.0.$$

We computed *H*(P(*win*)_*post*_) separately for every level of information availability (0–5 windows). For each level of information availability, *i*, we considered all 2^*i*^ possible permutations of winning and losing stimuli. For each permutation, we computed the binomial probability that the winning stimulus would be in the majority in the final sequence:

$$\textrm{P}\left( win|i,{w}_{min}\right)=\left\{\ \begin{array}{c}{\sum}_{k={w}_{min}}^{5-i}\left(\genfrac{}{}{0pt}{}{5-i}{k}\right){0.5}^{5-i},\kern0.75em \exists {w}_{min}\\ {}\kern5.75em 0\kern2em ,\kern4.25em \nexists {w}_{min}\end{array}\right.$$

where *i* is the number of informative windows, and *w*_*min*_ is the minimum number of additional winning stimuli required for a majority, if it exists (if it is impossible to win (∄*w*_*min*_), P(*win*) = 0). For each permutation, we derived the corresponding *H*(P(*win*)_*post*_) and information content, *I*. We then averaged *I* across all possible permutations for each level of information availability and used these values as the “Information Value” corresponding to those levels.

As an example, when three informative windows were available (*i* = 3), we considered each of the eight (2^3^) possible permutations of winning and losing stimuli that could possibly be displayed on those windows. We computed the probability that each three-window permutation could result in a win, based on the possible permutations in the remaining two (5 – *i*) windows. We derived the corresponding entropy *H*(P(*win*)_*post*_) and information content *I* for each permutation using the above formulae, and then averaged *I* across all 2^3^ permutations to derive the mean information content offered by three informative windows.

#### Model 4—Information increases the probability of earlier resolution of uncertainty

A final model defined Information Value as the probability that the informative windows could definitively resolve uncertainty in advance of the final outcome. This was computed as the binomial probability, *P*(*early*), that either a winning or a losing outcome could be known in advance based on the number of informative windows, *i*. This represented the proportion of the total 2^*i*^ possible permutations that could resolve uncertainty prior to feedback (i.e., contained at least three winning or losing stimuli).

$$P\left( early|\ i\right)=\left\{\kern0.5em \begin{array}{c}{\sum}_{k=3}^i\left(\genfrac{}{}{0pt}{}{i}{k}\right){0.5}^{i-1},\kern1.75em i\ge 3\\ {}\kern4em 0,\kern4.25em i<3\end{array}.\right.$$

As an example, when three informative windows were available (*i* = 3), only two of the eight (2^3^) possible permutations could reveal a definitive outcome (three winning stimuli or three losing stimuli), resulting in *P*(*early*| *i* = 3) = 0.25.

Models were fit using the glmer function in R (Bates et al., 2015), and fits were compared by computing the Bayesian information criterion (BIC; Table 1).

#### Results and discussion

Model comparisons revealed that behavior was best fit by the model postulating that decisions were influenced by the probability of earlier resolution of uncertainty (Model 4). This model outperformed the next best-fitting model (Entropy Reduction, Model 3) by 40 units. We tested goodness-of-fit with 1,000 bootstrapped simulations, and confirmed that early resolution of uncertainty was a significant predictor of choice (99% confidence intervals, [1.07; 1.38]). In a supplementary analysis, we also considered the possibility that each of Models 2–4 partially contributed to choice (Supplementary Material). We included Information Value from each of Models 2–4 in a single model to examine which factor served as the best predictor for choice. Although each model significantly contributed to behavior, Model 4 had the greatest effect. Together, these results suggest that information availability influenced risk-based decisions primarily based on its capacity to definitively resolve uncertainty in advance of the final outcome being declared.

## General discussion

Here, we provide a strong test of the capacity of non-instrumental information to influence decision-making under risk. Our data reveal three key findings. First, information availability consistently increased risk preference, even though that information could not possibly alter future outcomes. Second, this effect generalized across multiple levels of perceived risk. Finally, choices were best described by a model where the value of information was based primarily upon its capacity to sooner resolve uncertainty about the final outcome. Overall, these results show that decision-making under risk is significantly impacted by factors other than the statistical distributions of the possible outcomes, and highlight the importance of anticipatory information to risk-based decisions.

In this study, we examined whether non-instrumental information can modulate a primary reward-based decision that should be governed by more motivationally salient factors. This contrasts with the typical approach in the field, which is to explicitly probe the value that individuals ascribe to non-instrumental information. Our results provide clear evidence that information availability increased the preference for risky behavior, despite that information having no possibility of altering outcomes (cf. Falk & Zimmermann, 2016; Ganguly & Tasoff, 2017; Masatlioglu et al., 2017; Nielsen, 2020). Importantly, we found that the effect of information availability persisted even when we increased the motivational salience of the risk involved in each choice. This is in keeping with previous studies indicating that reward magnitude and outcome variance may play less critical roles in driving information preference (Kobayashi & Hsu, 2019; Liew & Newell, 2021). Importantly, our result builds on this earlier work, by showing that this effect extends to the case when the primary decision is not about the information itself.

Our study also tested the prediction that any information capable of reducing or resolving uncertainty should be valued, even if it is incomplete. Until now, there have been few direct tests of this prediction, as most studies have delivered information in an all-or-nothing manner (Bennett et al., 2016; Brydevall et al., 2018; Goh et al., 2021; Iigaya et al., 2020; Jezzini et al., 2021; Kobayashi & Hsu, 2019; Lau et al., 2020; Liew et al., 2022; van Lieshout et al., 2018). Although some studies have varied the probabilities with which an informative outcome might be delivered, such information (if delivered) definitively resolved any remaining uncertainty (e.g., Bromberg-Martin & Hikosaka, 2011; Charpentier et al., 2018). It is rare for a study to test individuals’ desire to view incomplete information about a reward outcome (e.g., Cabrero et al., 2019). Here, in directly testing the utility of partial information, we in fact find evidence that the capacity of information to partially reduce uncertainty did not monotonically increase gamble preference.

Instead, individuals ascribed greater value to having no information whatsoever, than partial information that had no potential to reduce uncertainty. This was reflected by the small, but significant, preference to gamble in the complete absence of information, versus when one informative window was available. This offers a counterpoint to the idea that any information that reduces uncertainty should be valuable. Broadly, it appears similar to the “ostrich effect” in behavioral finance, in which individuals prefer to avoid information about particular outcomes, particularly if it may evoke a negative emotion (Gul, 1991; Karlsson et al., 2009; Zhu et al., 2017). Prima facie, this should not apply to our study, given that win/loss outcomes were equiprobable. However, potential losses are known to loom larger than gains (Kahneman & Tversky, 1979), and the potential for a negative outcome may have led to a preference to remain completely ignorant, rather than obtain necessarily incomplete knowledge that could only evoke a sense of dread. Obviously, however, this effect needs to be replicated and clarified in future work.

The effect of non-instrumental information on choice appeared to be driven predominantly by its potential to definitively resolve uncertainty, over its capacity to incrementally reduce any degree of uncertainty. These two constructs are typically not clearly distinguished, despite being separate constructs (e.g., Bromberg-Martin & Monosov, 2020; FitzGibbon et al., 2020; Gottlieb et al., 2020; Gottlieb & Oudeyer, 2018; van Lieshout et al., 2020). Our findings that choices were driven primarily by the early resolution of uncertainty is consistent with findings in behavioral economics that individuals prefer to resolve uncertainty sooner rather than later (Brown & Kim, 2014; Kocher et al., 2014; von Gaudecker et al., 2011; Zimmermann, 2015), although this has previously been shown mostly for instrumental information.

In addition to advancing our understanding of information preference, our data also build on traditional models of decision-making under risk. These models typically focus on the statistical distributions of payoffs. They describe to varying extents how the subjective utility of an option is subject to an individual’s risk attitude and frame of reference (Holt & Laury, 2002; Weber, 1994), but do not typically consider the events that unfold between decision-making and outcomes. In contrast, our results suggest that the opportunity to obtain post-decisional knowledge about one’s choices, even if it cannot alter future outcomes, impacts on value-based decisions. This challenges future models of risky decision-making to incorporate the impact of factors irrelevant to estimates of risk preference. One approach may be to combine existing models with those that emphasize the utility of anticipated outcomes (Berns et al., 2006; Iigaya et al., 2020; Loewenstein, 1987; Story et al., 2013).

In our design, it was critical to ensure that any effects of information availability were not simply due to the reject option being nominally less perceptually stimulating (or more ‘boring’) than the alternative. To that end, we designed the task such that the reject option comprised the identical sequence of neutral faces for all experimental conditions. This ensured that the reject option could not have a differential effect on any given level of information. Our analyses demonstrate the efficacy of this approach. In particular, we found that choices were best modelled by a very specific preference for earlier resolution of uncertainty, which is highly unlikely to have arisen simply from overall differences in how the reject and accept options were perceived. These data suggest that our novel design offers a sensitive approach to probe the effect of information on risk-based choice.

Finally, we note that gamble rates in our study were relatively high, given previous findings that people tend to reject mixed gambles with symmetrical odds (e.g., Brenner et al., 2007; Inesi, 2010; Schmidt & Zank, 2005). However, we note that such findings are not ubiquitous (e.g., Rozin & Royzman, 2001; Yechiam & Hochman, 2013), and the tendency to reject such gambles can be made to disappear, or even reverse (e.g., Birnbaum & Bahra, 2007; Ert & Erev, 2008; Koritzky & Yechiam, 2010). In our study, several factors may have contributed to increased risk-seeking behavior, including: the provision of a modest endowment (Birnbaum & Bahra, 2007; Thaler & Johnson, 1990; Tversky & Kahneman, 1992); the equal probability of wins and losses that were a relatively small proportion of that endowment; the inclusion of all gambles in the payout calculation; and participants’ understanding that they would not leave the experiment in debt. Regardless, the high acceptance rate does not impact our main finding on the effect of information on risky decisions.

In summary, this work highlights the capacity of non-instrumental information to influence decision-making under risk. We provide a direct empirical test of several important predictions from a growing body of work on non-instrumental information, particularly with respect to its utility, and the value of partial information. Our data also provide an important counterpoint to extant models of decision making under risk, which focus on the statistical properties of outcomes and payoffs. Although these theories have been highly influential, they tend not to consider events that occur between decisions to act, and the delivery of the final outcome. Our findings suggest that these frameworks will benefit from considering the effect of anticipatory utility on choice behavior, particularly in the post-decision phase, even though it is of no instrumental utility to the individual.

### Supplementary Information

ESM 1 (DOCX 48 kb)
