# Supplementary material for: The availability of non-instrumental information increases risky decision-making
Source: Psychon Bull Rev. 2023 Apr 10;30(5):1975–87. doi: 10.3758/s13423-023-02279-1 (PMC10716073; doi:10.3758/s13423-023-02279-1)
Supplement: Supplementary file 1 — (DOCX 48 kb) [file 13423_2023_2279_MOESM1_ESM.docx]

# Supplementary Material

### Pooled Analyses

A logistic mixed model analysis on the pooled data reiterated the pattern of results for Information Availability in each individual experiment. The main effect of Information Availability was significant (χ^2^(5)=525.16, p<.001). Participants accepted gambles significantly more often in the zero-information condition (*M*=.63, *SEM*=.02) than when one informative window was available (*M*=.58, *SEM*=.02; z=-3.39, p_holm_=.003). Participants accepted slightly more gambles when zero vs two informative windows were available (*M*=.60, *SEM*=.02), but this was only significant at uncorrected thresholds (z=-2.16, p_uncorrected_=.031, p_holm_=.092). The remaining pairwise comparisons revealed the same effect as detailed in previous experiments. The likelihood of gambling was similar between the one and two window conditions (*M*=.60, *SEM*=.02; z=1.23, p_holm_=.436); increased as information increased from two to three windows (*M*=.72, *SEM*=.02; z=8.86, p_holm_<.001), and three to four windows (*M*=.80, *SEM*=.02; z=6.34, p_holm_<.001), before plateauing between four and five (*M*=.80, *SEM*=.01; z=-0.11, p_holm_=.91).

To investigate the overall relationship between Information Availability and Stake we pooled data from Experiments 2 and 3. These experiments were selected because information and stake were manipulated orthogonally. The main effect of Stake was significant and consistent with risk averse behavior (χ^2^(4)=148.26, p<.001). Lower stake gambles (e.g., 10¢, *M*=.74, *SEM*=.04; 20¢, *M*=.73, *SEM*=.03) were accepted more often than those with higher stakes (30¢, *M*=.65, *SEM*=.02; 40¢, *M*=.60, *SEM*=.03; 50¢, *M*=.63, *SEM*=.03; all p_holm_<.001). As in Experiments 2 and 3, the interaction between Information Availability and Stake was not significant (χ^2^(20)=19.87, p=.47; ***Fig. 4b***). Together, these results provide further confirmation of the effect of non-instrumental information on risky decisions, and indicate that this effect generalizes across different levels of perceived risk.

### Participants were engaged throughout each experimental session

To ensure that the effects of Information Availability on risky decisions were not confounded by participants becoming disengaged over time, we performed an additional analysis to confirm that these effects were stable across each experimental session. We repeated the mixed model analyses from each experiment (and the pooled data), and included Trial Number (and all of its interactions) as additional fixed effects. In all analyses, Trial Number had a small but significant main effect, such that individuals accepted slightly fewer gambles over time (Experiment 1: β=-.003, χ^2^(1)=16.61, p<.001; Experiment 2: β=-.0002, χ^2^(1)=9.24, p=.002; Experiment 3: β=-.0005, χ^2^(1)=6.72, p=.010; pooled data: β=-.0002, χ^2^(1)=29.66, p<.001). Critically, however, inclusion of Trial Number did not change the results for the remaining fixed effects. Furthermore, Trial Number did not significantly interact with Information Availability (p-values for all experiments >.15). These analyses confirm that the effect of Information Availability on risky decisions was stable over the course of each experiment despite a general propensity for participants to accept fewer gambles over time.

In addition, participants showed evidence of appropriate task engagement across all three experiments, with timely and appropriate responses on almost every trial. Participants omitted a response on only 50 out of 12,780 trials across all three experiments. In addition, data indicate that participants were responsive to the main experimental manipulations. There was a strong and significant effect of Information Availability in three independent experiments (Experiment 1, χ^2^(5)=105.65, p<.001; Experiment 2, χ^2^(5)=243.07, p<.001; Experiment 3, χ^2^(5)=190.73, p<.001; pooled data, χ^2^(5)=525.16, p<.001). Similarly, the main effect of Stake was significant in both Experiments 2 and 3 (Experiment 2, χ^2^(4)=153.16, p<.001; Experiment 3, χ^2^(4)=20.25, p<.001; pooled data, χ^2^(4)=141.01, p<.001). Together, these data indicate that participants were appropriately engaged in all tasks.

### The results are preserved even when excluded participants were included in the analysis

Of the 77 participants recruited, six were excluded following post-experiment debriefs, in which they reported believing that the odds of winning were not fixed (*n*=0 in Experiment 1; *n*=4 in Experiment 2; *n*=2 in Experiment 3). To investigate the consequences of doing so, we repeated all analyses on the entire cohort of *N* = 77. Importantly, this did not alter the pattern of results for any of the analyses reported in the main text. With regards to our main finding, the main effect of Information Availability remained significant in the pooled data of 77 participants (χ^2^(5)=512.68, p<.001). Notably, in this analysis, participants were still more likely to accept gambles in the zero information condition vs when one informative window was available (z=-3.486, p_holm_=.002).

### Supplementary Modelling

Although Model 4 offers the most parsimonious explanation for the data, it is possible that Models 2 and 3 may have also partially influenced behavior. To investigate this possibility, we fit a logistic mixed model, in which Information Value comprised three additive factors derived from Models 2 to 4. Each model was included as a numeric variable with their corresponding Information Value, with subject-specific intercepts included as random effects. To examine the contribution of each model on decision-making, we compared the full model to a reduced model that excluded the factor of interest. As expected, excluding Model 4 (Early Resolution of Uncertainty) had the greatest effect on decisions (χ^2^(1)=90.33; p<.001). However, excluding Models 2 (Linear) and 3 (Entropy Reduction) also significantly impaired model fits, although to a lesser extent (Model 2, χ^2^(1)=49.25, p<.001); Model 3, χ^2^(1)=48.42, p<.001). This indicates that choices were indeed most strongly driven by the capacity of information for early resolution of uncertainty, but that individuals were likely influenced by other properties of information, such as its capacity to incrementally reduce uncertainty.
